# Stability evaluation of ZnO nanosheet based source-gated transistors

**DOI:** 10.1038/s41598-019-39833-8

**Published:** 2019-02-27

**Authors:** A. S. Dahiya, R. A. Sporea, G. Poulin-Vittrant, D. Alquier

**Affiliations:** 1 GREMAN UMR 7347, CNRS, Université de Tours, INSA-CVL, 16 rue Pierre et Marie Curie, 37071 Tours, France; 2 Advanced Technology Institute, Department of Electrical and Electronic Engineering, University of Surrey, Guildford, Surrey, GU2 7XH United Kingdom

## Abstract

Semiconducting nanostructures are one of the potential candidates to accomplish low-temperature and solution-based device assembly processes for the fabrication of transistors that offer practical solutions toward realizing low-cost flexible electronics. Meanwhile, it has been shown that by introducing a contact barrier, in a specific transistor configuration, stable device operation can be achieved at much reduced power consumption. In this work, we investigate both one-dimensional ZnO nanowires (NWs) and two-dimensional nanosheets (NSs) for high performance and stable nano-transistors on conventional Si/SiO_2_ substrates. We have fabricated two variant of transistors based on nanoscale single-crystalline oxide materials: field-effect transistors (FETs) and source-gated transistors (SGTs). Stability tests are performed on both devices with respect to gate bias stress at three different regimes of transistor operation, namely off-state, on-state and sub-threshold state. While in the off-state, FETs shows comparatively better stability than SGTs devices, in both sub-threshold and on-state regimes of transistors, SGTs clearly exhibits better robustness against bias stress variability. The present investigation experimentally demonstrates the potential advantages of SGTs over FETs as driver transistor for AMOLEDs display circuits which require very high stability in OLED driving current.

## Introduction

Over the last couple of decade, the scientific community has made large leaps in the development of large area high performance Thin-Film Transistors (TFTs)^1–3^, in particular as a backplane driver transistor in display technologies^4–6^. The field of TFTs has started and matured with silicon based technology using thin films of amorphous silicon (a-Si) and/or Low Temperature Poly-Silicon (LTPS) as active channel layer. However, the last decade observed a surge in the research and improvement activities for the development of TFTs and TFT applications based on advanced nanomaterials. The motivation behind these significant efforts and subsequent advances includes reducing the manufacturing cost, searching of high mobility material and finding a current source to drive organic light emitting diodes (OLED) displays where neither a-Si nor LTPS are ideal driver because of inferior stability^7^. There are different material technologies being investigated such as organic semiconductors^8^, thin film of metal oxides^9^ and nanostructure of various materials such as carbon nanotubes (CNTs), Si, ZnO, SnO_2_, In_2_O_3_, etc. for the development of high performance TFTs for various device applications^10^.

Stable TFT operation is a prerequisite for most applications, such as Active Matrix Organic Light Emitting Diodes (AMOLEDs) display pixel circuits. Stability of TFTs during operation remains one of the challenging issues to be addressed, since its first introduction in 1960s, for their effective implementation into device applications. The effect of gate-bias stress is one of the most studied stability issues for TFTs stable operation. General observation during the gate induced stress is that the threshold voltage (*V*_*TH*_) shifts towards positive or negative gate voltage (depending on positive or negative gate voltage applied). Variations in an on-current (I_ON_) of TFT can occur because of electrical stress induced by the long-time device operation. There are two mechanisms proposed in the literature for the observed threshold shift in TFTs. Specific to amorphous silicon (a-Si), one mechanism arises because of the motion of bonded hydrogen in the a-Si channel during prolonged gate bias-stress and creates extra defect sites in the channel^4,11^. These defects sites act as trap centers for charge carriers and cause in the reduction of TFT current^7^. The second mechanism for the shift in *V*_*TH*_ is common to all materials and is the transfer of mobile charges to immobile trapping states at the semiconductor/insulator interface^12^ or at the semiconductor/ambient interface^13^. This accumulated charge layer can effectively screen the gate voltage field and reduce its ability to control the channel, and thus, the drain current. These mechanisms, consecutively, lower the luminance of individual pixels over time, causing display non-uniformity.

Since both mechanisms are related to defects, either in semiconductor and/or in gate oxide, very little can be done about it in TFTs where the current is ‘channel controlled’. Although silicon oxide passivation of Indium-Gallium-Zinc-Oxide (IGZO) based TFTs showed negligible *V*_*TH*_ shift up to 10 h of continuous operation, it has surely added another processing step which will further increase the manufacturing cost of the device^14^. As another effective and potential solution to avoid the I_ON_ of TFT to decrease during operation, a new kind of field-effect transistor (FET) was introduced by Shannon and Gerstner, so-called “source-gated transistors (SGT)”^15^. The Schottky-barrier SGT device does not differ significantly from conventional TFTs and/or FET except for the necessity for a Schottky barrier at the source and a drain contact which is preferred to be, but is not required to be, ohmic. Introduction of a Schottky barrier at the source contact leads to abrupt saturation in current-voltage characteristics, even at very high gate voltages, and remains stable with further increase of drain-source voltage^6,15–18^. That means SGTs are ‘contact controlled’ devices compared to conventional TFTs which are ‘channel controlled’ ones. Such abrupt current saturation in output characteristics of TFTs leads to many interesting consequences: (1) very large intrinsic gain, (2) low-power operation, and (3) stability under prolonged gate-bias stress.

The low-temperature and solution-based assembly of FETs, using semiconducting nanostructures, offer practical solutions toward realizing low-cost, flexible self-powered autonomous systems^19^. Meanwhile, employing many different semiconducting nanostructures, including Si nanowire (NW) arrays^20^, ZnO NWs^21^ and ZnO nanosheets (NSs)^16^, at near room temperature (RT) device assembly processes, low power consumption SGTs have been demonstrated in the past. Such fabrication process characteristics, coupled with flat output current saturation features of SGTs, are ideal for a range of low power applications, including wearable electronics and self-powered systems. In the present work, we will first investigate two different ZnO nanostructures, namely nanowires (NWs) and nanosheets (NSs), as an active semiconducting channel, for the fabrication of stable FETs with an ohmic contacts. Once the channel material (ZnO NS) is locked, a comparative study on the device stability of SGTs and FETs, based on ZnO NSs, will be presented.

## Experimental Data

### Growth of ZnO NWs and NSs

Both ZnO nanowires (NWs) and nanosheets (NSs) are grown using Vapor-Liquid-Solid (VLS) growth mechanism. The VLS growth of ZnO NWs and NSs is performed inside a horizontal quartz tube furnace by carbothermal reduction of ZnO nanopowder on c-plane and r-plane sapphire substrates, respectively. Prior to ZnO nanostructure synthesis, cleaned sapphire substrates were coated with a Au film (2 ± 1 nm) using a electron-beam evaporator. Au Coated substrates and the source material (ZnO and C at 1:1 weight ratio) were placed on top of an alumina ‘boat,’ which is inserted inside a furnace. An Ar ambient was maintained inside the growth chamber throughout the whole process. To initiate the growth, the furnace was ramped up to 850 °C for NW and 875 °C for NS formations, with a fixed ramp rate of 30 °C min^−1^ and a growth time at the plateau of 180 min. After the growth, the furnace was switched off and left to cool naturally to room temperature and growth substrates were recovered thereafter. See ref.^22–24^. for more details on growth and characterization of nanomaterials.

### Fabrication of NW- and NS based transistors

To fabricate the ZnO SGT/FET devices, the as-grown nanostructures (NW and/or NS) were dispersed onto highly doped p ^++^−Si substrate with 170 or 290 nm thick thermally grown SiO_2_ layer. Using electron-beam lithography, source and drain (s/d) contacts were defined on to opposite ends of a selected ZnO nanomaterial. Standard metallization and lift-off fabrication protocols were carried out for s/d metal deposition. All electrical assessment of the fabricated SGTs/FET were carried out using a Cascade Microtech Summit 11k probe station with single source measure unit (2636 A by Keithley Instruments) under dark ambient conditions. See refs.^16,22,24^ for more details.

## Results and Discussions

### ZnO nanostructures based FETs comparison

**Figure 1:**
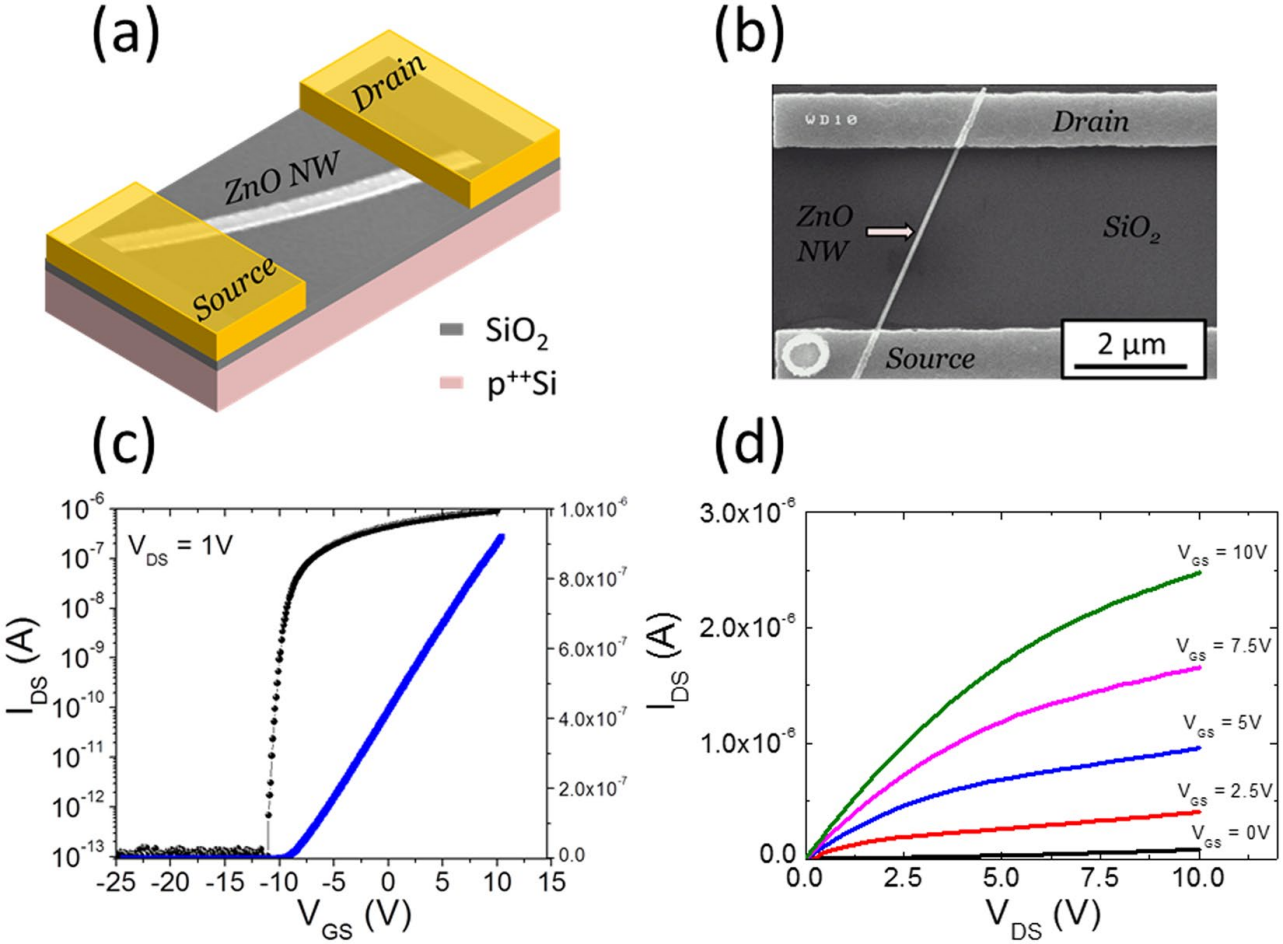
The electrical transport characteristics attained by a representative single NW-FET device with channel length L of ~5 µm and diameter d of ~100 nm: (**a**) schematic/SEM image, (**b**) SEM image, (**c**) I_DS_-V_GS_ transfer scan linear and log curve measured at V_DS_ = 1 V, and (**d**) the corresponding I_DS_-V_DS_ output scan curves varying the V_GS_ from 0 to 10 V with a step of 2.5 V.

**Figure 2:**
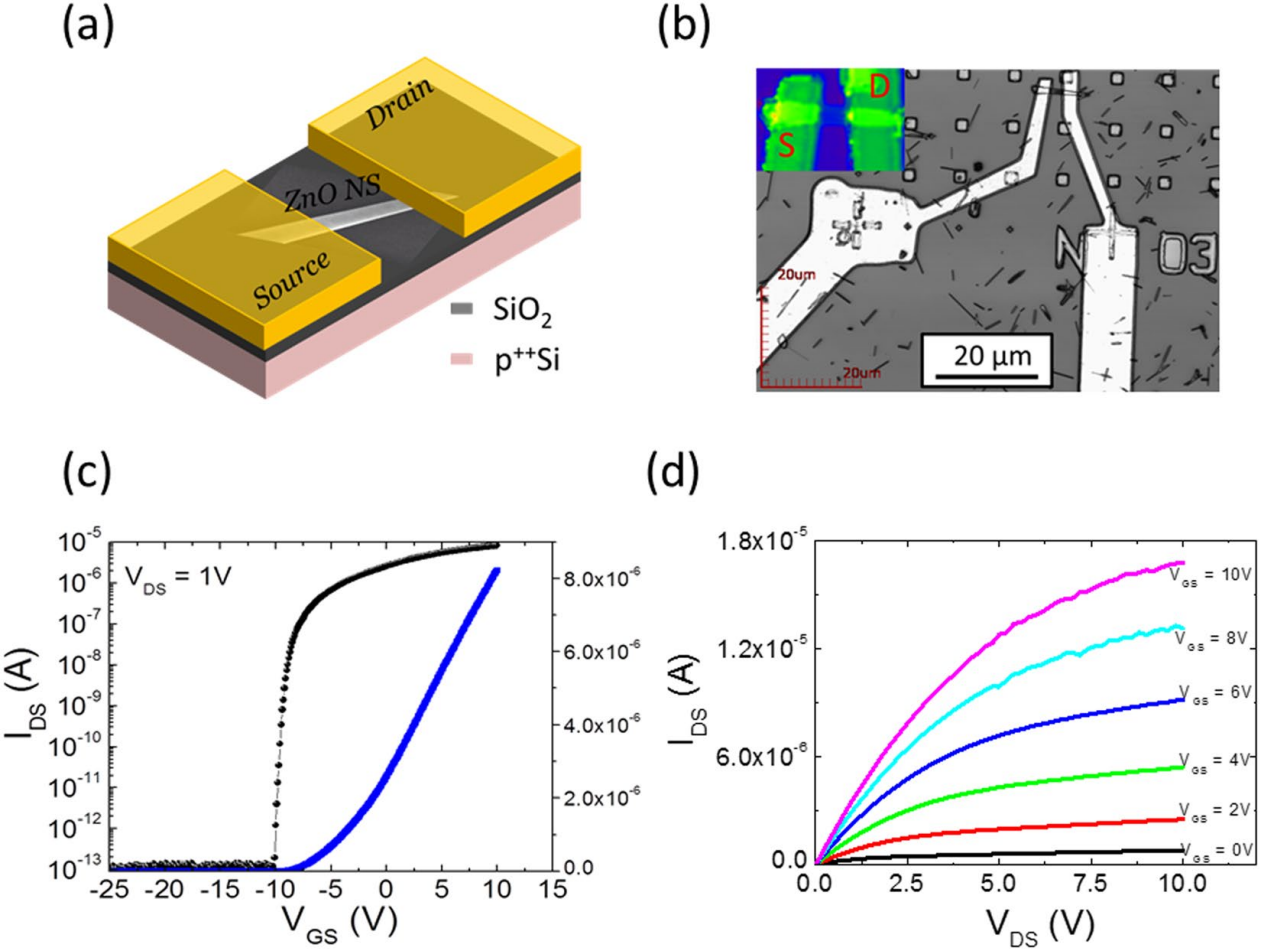
The electrical transport characteristics attained by a representative single NS-FET device with channel length L of ~2.5 µm and width W of ~1.5 µm: (**a**) Schematic/SEM image, (**b**) optical image, (**c**) I_DS_-V_GS_ transfer scan linear and log curve measured at V_DS_ = 1 V, and (**d**) the corresponding I_DS_-V_DS_ output scan curves varying the V_GS_ from 0 to 10 V with a step of 2.5 V.

Performance comparison of ZnO NW- and NS-FETs is made by measuring I-V characteristics of both types of FET devices in identical conditions. To obtain the transfer scans, the gate-source voltage (*V*_*GS*_) is swept from −25 V to +10 V at a drain-source bias (*V*_*DS*_) of 1 V. The families of output scans are obtained by sweeping *V*_*DS*_ from −10 V to 10 V (only positive *V*_*DS*_ voltages shown) and *V*_*GS*_ is incrementally stepped from 0 V to 10 V, after a full sweep of *V*_*DS*_. Figure 1 shows a device schematic, its Scanning Electron Microscopy (SEM) associated image and the transfer and output scans of typically constructed NW-FET devices. Similar results obtained for NS-FET devices are shown in Fig. 2. From these experimental output data obtained for both type of devices, it can be seen that increasing *V*_*GS*_ towards positive values resulted in an increase of the drain current (*I*_*DS*_). This device behavior suggests a n-channel accumulation-type FET. The observed increase in *I*_*DS*_ with incremental positive increase of *V*_*GS*_, in the output scans, also confirms the n-channel behavior exhibited by both types of devices.

#### FET key performance parameters include

the off-state current (I_OFF_), on-state current (I_ON_), on/off current ratio (I_ON/OFF_), sub-threshold swing (*s-s*) and field-effect mobility (*μ*_*FE*_). From the semi-log plot of the transfer scan for NW- and NS-FET, the extracted values of I_OFF_, I_ON_, I_ON/OFF_ ratio, *s-s* parameter and *µ*_*FE*_ are shown in Table 1 (average data from 8 separate devices). The μ_FE_ parameter is evaluated from the standard MOSFET model of a FET operating in the linear regime using Eq. 1 for the NW-FET, while the same parameter for NS-FET is obtained using Eq. 2^16,21^:

1 $${\mu }_{FE}={g}_{m}\frac{{L}^{2}}{{V}_{DS}{C}_{NW}}$$

2 $${\mu }_{FE}=\frac{L}{W}\frac{{g}_{m}}{{V}_{DS}{C}_{NS}}$$

where g_m_ is the transconductance, L is the channel length, W is the channel width and *V*_*DS*_ is the applied drain source voltage. The gate-oxide capacitance for NW-FET (*C*_*NW*_) and surface capacitance of NS-FET (*C*_*NS*_) can be expressed as follows:

3 $${C}_{NW}=\frac{2\pi {\varepsilon }_{0}{\varepsilon }_{ox}L}{cos{h}^{-1}(\frac{{r}_{NW}+{t}_{ox}}{{r}_{NW}})}$$

4 $${C}_{NS}=\frac{{\varepsilon }_{0}{\varepsilon }_{ox}}{{t}_{ox}}$$

where *ε*_0_ is the free space vacuum permittivity (8.85 × 10^−12^ F/m), *r*_*NW*_ is the NW radius and *ε*_*ox*_ and *t*_*ox*_ are relative dielectric permittivity and thickness of SiO_2_ (~300 nm), respectively. The calculated value of *C*_*NW*_ is ~0.4 fF while the *C*_*NS*_ value is 1.15 F/m^2^.

**Table 1:** Comparison between ZnO NW-FET and NS-FET based on the extracted key performance metric parameters for FETs under similar bias conditions.

Type of FET	No. of devices	Mobility (cm^2^/Vs)	Subthreshold swing (mV/dec)	ON/OFF current ratio	*V*_*TH*_ range
NW-FET	8	11 ± 9	500 ± 150	~10^7^	−9 to 9 V
NS-FET	8	95 ± 20	400 ± 150	~10^8^	−9 to 5 V

**Figure 3:**
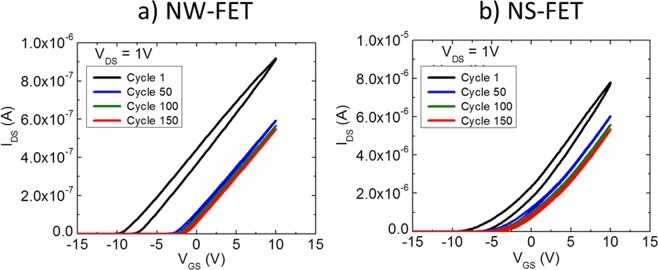
I_DS_-V_GS_ transfer characteristics showing stability test of fabricated devices up to 250 min of continuous operation for (**a**) NW-FET, and (**b**) NS-FET.

It can be seen from Table 1 that the performance of ZnO NS-FET is comparable with the ZnO NW-FET in almost all performance parameters except for the µ_FE_ values which are higher for NS-FET devices. This is expected since the contact area of NS-FET devices is larger than that of NW-FETs, resulting in higher injection of charge carriers and thus, higher transconductance values. Next, in order to take the performance comparison further, electrical-bias stress dependent stability for both NW and NS-FET devices are performed under identical conditions. Indeed, electrical gate-bias stress is a serious issue that affects most of the FET devices including MOSFETs, TFTs and NW-FETs^14,25^. For bias-stress evaluation, we obtained *I*_*DS*_ by sweeping *V*_*GS*_ from −15 V to +10 V (forward voltage sweep) and from +10 V to −15 V (reverse voltage sweep) at fixed *V*_*DS*_ = 1 V. This full scan, from forward to reverse voltage sweep, is considered as one cycle of bias-stress. The value of the gate voltage sweep rate is fixed at 0.5 V/sec for the entire test. Figure 3 shows the results of the electrical gate-bias stress for NW- and NS-FET where the transfer scan is performed continuously up to 150 cycles (250 min).

**Figure 4:**
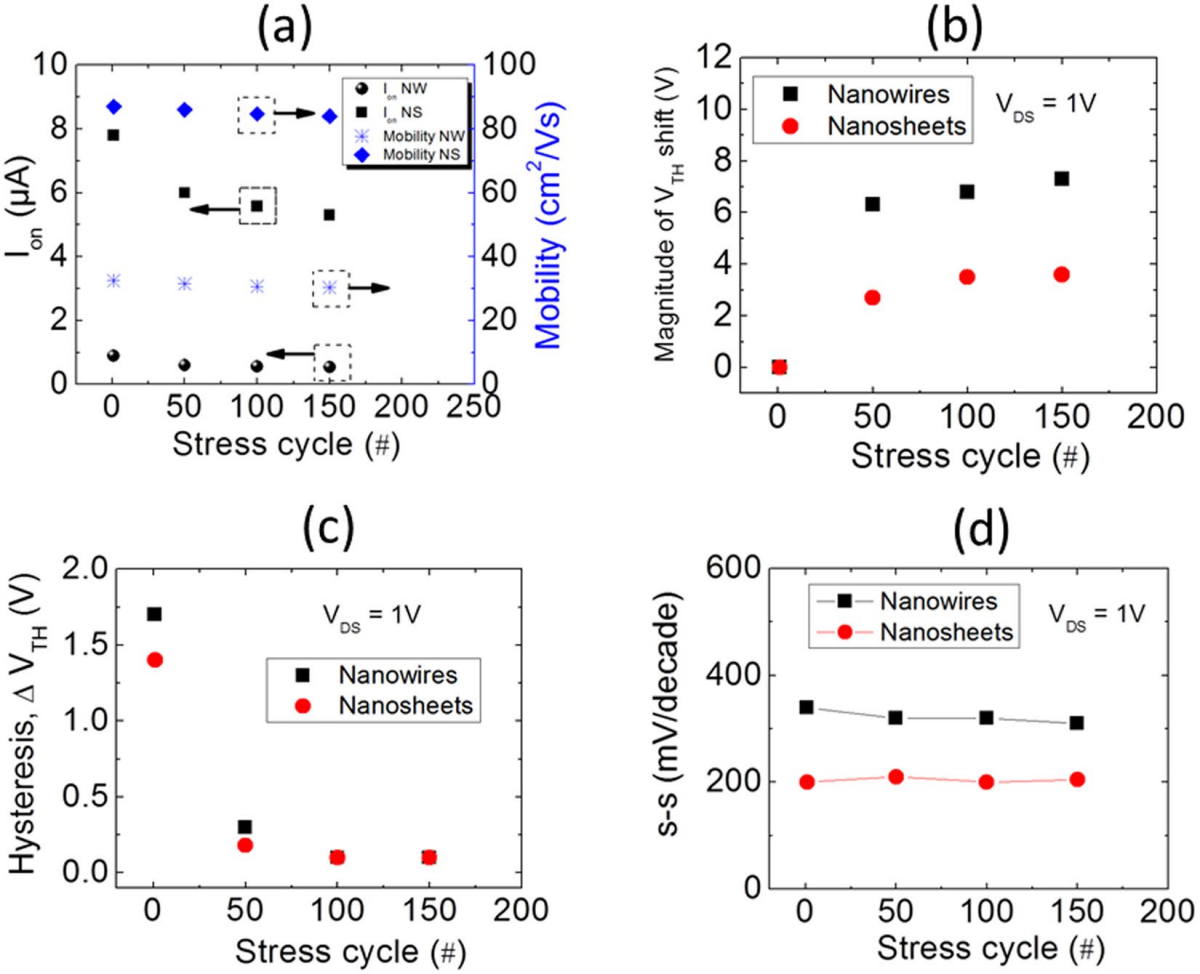
The effect of positive bias stress on NW- and NS-FET devices: (**a**) on-current and field effect mobility, (**b**) threshold voltage shift, (**c**) hysteresis and (**d**) sub-threshold swing.

Important information that can be derived from the bias-stress measurements are device on-current and mobility evolution, shift in threshold voltage, hysteresis progression and sub-threshold swing fluctuation with stress time. The results of these investigations are shown in Fig. 4. The first observation that can be made on these stress stability measurements, for both device types, is the decrease in the FET on-current with the increase in stress time. Consequently, the field-effect mobility of the devices decreases (Fig. 4a). For the complete understanding of the underlying mechanism of the I_ON_ decrease, we have also extracted the shift in threshold voltage (*V*_*TH*_), magnitude of hysteresis and *s-s* values with the stress time. It is evident, from Fig. 4b, that these devices showed positive threshold voltage shift with time. For n-type TFTs, this positive shift of *V*_*TH*_ with gate-bias stress can be well explained using charge-trapping models, as mentioned previously. To identify the exact charge-trapping mechanism, it has been shown that observing the *s-s* values, with stress time, can give an idea of the mechanism. While an increase in *s-s* value signifies generation of new charge-traps at the semiconductor-oxide interface, a constant *s-s* value represents charging and discharging of preexisting traps^14^. As can be seen from Fig. 4d, for both device types, the *s-s* value remains constant for the entire test. This clearly suggests that the observed *V*_*TH*_ shift in our devices is related to the filling of already present trap-states at the semiconductor/insulator interface. This increase of interface trap density with time results in the degradation of effective channel mobility and on-current of the devices. A quantitative estimation of the interface charge-trap density can be made by measuring the hysteresis of the devices. As can be seen from Fig. 4c, the ZnO NS-FET showed comparatively lower Δ*V*_*TH*_ and consequently, lower decrease in the on-current of the FET device.

With the electrical gate-bias results and from Table 1, it is fair to conclude that ZnO NS-FETs show slightly superior performances compared to ZnO NW-FETs. It is further to note that the major cause of I_ON_ degradation is related to the filling of already present trap-states at the semiconductor channel/insulator interface. Therefore, ZnO NSs are really promising structures for achieving stable on-current operation in contact-controlled SGTs.

### Electrical bias stability of ZnO NS-SGTs

**Figure 5:**
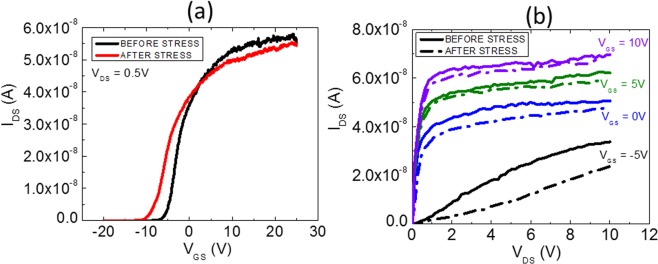
I-V characteristics of the SGT device before and after stress (**a**) transfer (**b**) output scan.

In our previous report, we have shown excellent field-effect transport behavior of ZnO NS-SGTs with abrupt drain current saturation at low drain voltages, well below 2 V, even at very large gate voltages^16^. In this work, we will evaluate the stability of SGT devices under prolonged electric bias stress. Figure 5 shows the transfer and output characteristics of a typical ZnO NS-SGT device before and after the gate-bias stress. It is to note that the applied stress conditions are similar to the one tested for conventional FETs in the last section (transfer scans performed continuously up to 150 cycles with sweep rate of 0.5 V/sec). As can be noticed from the I-V results, the SGT device shows very good stability under extreme gate-bias stress. The *V*_*TH*_ shift during the SGT operation, is also very low and so is the decrease in the on-current of the device. From the transfer scan of the SGT device, we evaluated following performance metrics for NS-SGT device: n-channel normally-on transistor with threshold voltage of −5.3 V, I_ON_ (~56 nA)/I_OFF_ (~10 fA), current ratio of ~10^5^, sub-threshold swing value of ~1.3 V/decade, and field-effective mobility or effective mobility, at room temperature of approximately 5 cm^2^/Vs. The output characteristics (Fig. 5b) show early current saturation which occurs specifically through source pinch-off. The lowest curve in the figure is in a low-V_GS_ regime where the channel is less conductive than the source region (assumed weak accumulation) so the whole device behaves like a FET at low V_GS_, with a typical saturation voltage and saturated current behavior^18^.

**Figure 6:**
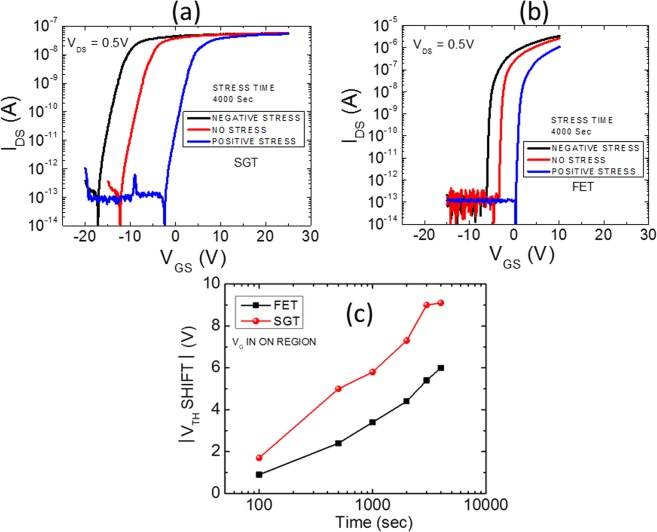
The evolution of the transfer curve with gate-bias stress for: (**a**) SGT and (**b**) FET. (**c**) Extracted magnitude of V_TH_ shifts with stress time for both SGT and FET with PGBS.

Next, we have studied the evolution of the transfer scan with gate-bias stress by applying both positive and negative drain-source bias. In this case, we have interrupted the gate bias stress at fixed interval of time and then, measured the transfer scan again. For the entire series of transfer scans, the drain bias was fixed to 0.5 V while the gate bias was scanned from −20 to +25 V. In order to compare the magnitude of *V*_*TH*_ shift for SGT device, a similar device was fabricated with ohmic contacts (conventional FET) where the device current is dominantly controlled by the semiconductor channel. The conventional FET has been also tested under similar conditions. The results for both type of devices are presented in Fig. 6a,b whereas Fig. 6c shows the extracted magnitude of *V*_*TH*_ shift for both SGT and FET devices. It can be seen, from Fig. 6a,b, that in both SGT and FET devices the trend of *V*_*TH*_ shift is similar. For Positive Gate-Bias Stress (PGBS), the shift in *V*_*TH*_ is towards positive side while for Negative Gate-Bias Stress (NGBS), the shift is towards negative *V*_*GS*_. It is also interesting to note that there is no change in the s-s values of the devices with gate-bias stress (positive or negative).

**Figure 7:**
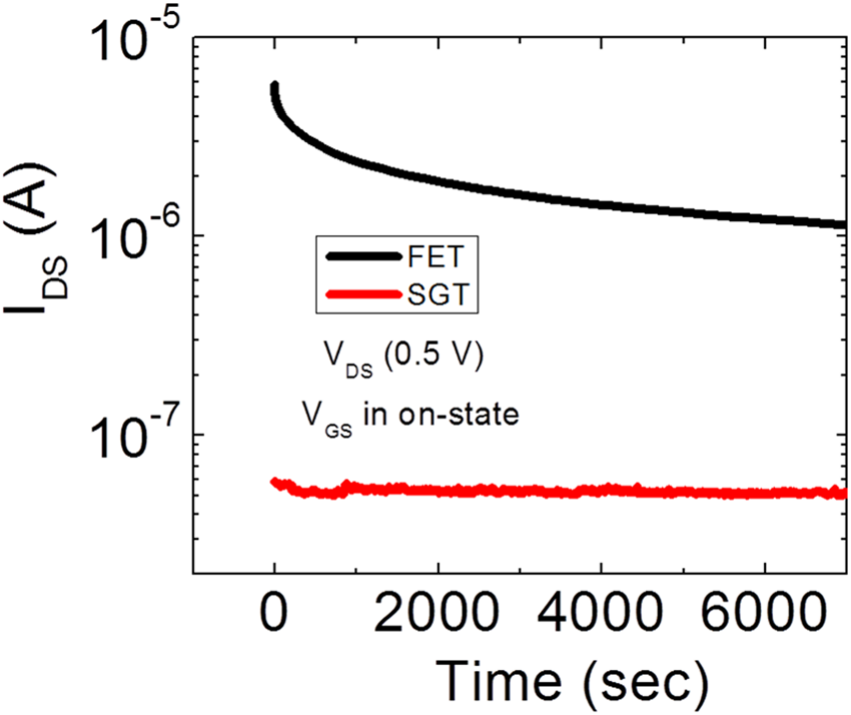
The drain current through the SGT and FET devices during the continuous stress applied for more than 100 min.

As previously mentioned, a positive *V*_*TH*_ shift in oxide TFTs under PGBS can be explained by two models: charge trapping or defect creation. While the parallel shift in *V*_*TH*_ without significant change in the s-s value during stress time is attributed to simple charge trapping in the gate dielectric and/or at the channel/dielectric interface, the positive shift in *V*_*TH*_ accompanying the change in s-s comes from the creation of defects such as oxygen vacancies within the oxide semiconductor channel material^26^. As the s-s value (1.3 V/dec) remains constant with the gate-bias stress, it can be concluded that *V*_*TH*_ shift is entirely due to a simple charge trapping in the gate dielectric and/or at the channel/dielectric interface. However, SGT devices also show significant *V*_*TH*_ shift with the PGBS. The device on-current is completely dominated by the contact and not by the channel. However, the subthreshold and off-states are generally controlled by the conductance of the parasitic channel formed between source and drain. Therefore, it is interesting to investigate the mechanism for *V*_*TH*_ shift for SGT devices. To do so, we have performed gate bias stress for the different functioning regimes of the transistor: namely on-state, off-state and sub-threshold regimes, for both types of devices. The results are shown in Fig. 7.

#### SGT on state (on-current)

Figure 6c shows the time dependence of *V*_*TH*_ shift for both SGT and FET devices under the application of a constant drain bias of 0.5 V. As can be seen from this Fig. 6c, the magnitude of *V*_*TH*_ shift with PGBS is larger in SGT devices. Next, we performed the gate bias stress at the different regimes of the transistor. First, we will discuss the on-state of the device. Figure 7 shows the gate bias stress in an on-state of the device by applying 25 V_GS_. It can be seen from Fig. 7, in an on-state of transistor regime, SGTs have significantly more robust behavior than FETs. It can be noted here that SGTs show only 7% decrease in I_ON_ compared to FETs which show more than 52% decrease in an on current for operation up to 2 hours. These observations can be well explained by understanding the charge transport mechanism in the on-state of the SGT device. In general, charge transport across the Schottky barrier (SB) is well explained using thermionic emission (TE) model^16^. However, TE charge transport model predicts that the SB height is independent of the reverse bias voltage which is not true in the present case. Here, thermionic theory does not account for the charge transport because of quantum mechanical tunneling and/or through the localized surface states and image force barrier lowering^16^.

As already mentioned, given the condition *V*_*GS*_ ≥ *V*_*TH*_ and *V*_*DS*_ ≥ *V*_*DS*_^*SAT*^ (output drain current saturation voltage), which are the conditions to be in on-state and pertain to the present case, the charge transport mechanism is controlled by thermionic field emission (TFE)^16^. The barrier lowering is such case is given by^27^,

5 $${\rm{\Delta }}{\O ^{\prime} }_{b}=\alpha E$$

where E is the gate field and α the effective barrier lowering constant. As the device on current, in such circumstances, is dominantly controlled by the SB and not by the channel, the effect of the interface traps, which are the main cause for the I_ON_ degradation, is negligible. Moreover, the current injection at the SGT source is a two-dimensional problem, but whichever of the two modes of operation is dominant^28^, the drain current is almost exclusively controlled by the source region. Thus, in the on-state of the device, SGT show very high stability compared to the FET one that is entirely controlled by the semiconductor channel.

#### SGT in s-s and off state

**Figure 8:**
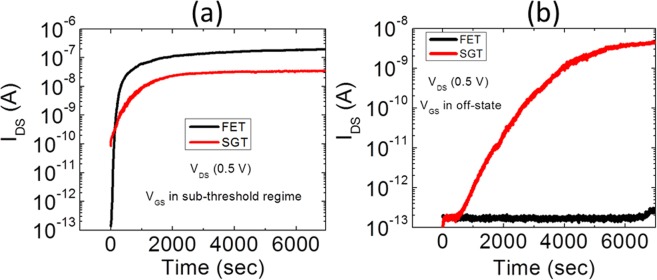
The drain current through the SGT and FET devices during the continuous stress applied for more than 100 min: (**a**) in s-s regime, and (**b**) off-regime of transistor.

The results for the gate bias stress in the s-s and off-state of the SGT device are shown in Fig. 8. As can be seen from Fig. 8a, in the subthreshold region, both devices show a similar trend of increasing drain current with the bias stress time. Although there is one order of magnitude difference in the drain current, the trend followed by both devices is similar. For the off-state of the devices, as shown in Fig. 8b, FET completely outperformed SGT device by demonstrating a very stable off-state current up to 2 h while there is a huge increase in the off-state current for SGT device. These observations are explained as follows:

Another factor dictating current transport characteristics in the SGT devices is the “parasitic” FET which is controlled by the semiconductor channel^18^. As previously mentioned, the only conditions at which output current is controlled by the SB is that *V*_*GS*_ ≥ *V*_*TH*_ and *V*_*DS*_ ≥ *V*_*DS*_^*SAT*^. However, if the applied *V*_*GS*_ is lower than the *V*_*TH*_ of the device, the current is dominantly controlled by the “parasitic FET” channel. This implies that, in the off-state, the gate field is not sufficient to start acting on the source SB and so, the charge transport is mainly governed by the thermionic emission over the source barrier, which is in series with the highly resistive semiconductor (in the absence of the channel accumulation) between the source and drain contacts. As the applied V_GS_ (−9 V) for bias stress in s-s region is less than the *V*_*TH*_, both devices show similar trends as both are controlled by the semiconductor channel. However, observing the bias stress in the off-state of both devices, we can see two completely different trends of output current with the increase of stress time. In Fig. 8b, the off-current in SGT starts to rise just after 300 sec whereas FET showed a very stable response. This rise of output current in the off-state of SGT devices could be explained as following: Fig. 6 shows a large negative threshold shift with NGBS, and what was considered “off current” may now be subthreshold. The off and subthreshold regions of SGT operation are generally governed by the properties of the weekly accumulated channel.

### Temperature dependence stability of SGTs

**Figure 9:**
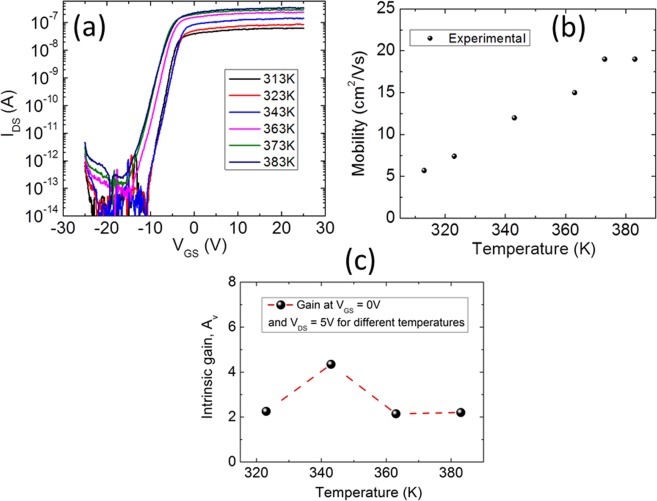
Temperature dependent transfer characteristics of ZnO NS-SGT device: (**a**) I_DS_-V_GS_ curve of the SGT device at V_DS_ = 1 V for various temperatures, (**b**) Mobility versus temperature plot shows increase in mobility with temperature for SGT device, and (**c**) temperature dependent intrinsic gain.

It can be argued that because the current in SGTs is controlled by the potential barrier present at the source contact and the device current is thermally activated, the saturation voltage characteristics of the device may change with thermal fluctuations, resulting into poor device performances at higher temperature. However, it has been shown, both by experimental and simulation works^29,30^, that the temperature dependence of the SGT devices can be controlled by careful device engineering while maintaining the obvious advantages of SGTs such as low saturation voltage and high output impedance in saturation. In this section, temperature dependence of SGT performance parameters, such as mobility and internal gain, is investigated for the device showed above. It is to note that the precise values of carrier mobility in SGTs are not essential as current is regulated at the metal-semiconductor (MS) interface and not by the source and drain separation (channel length). However, temperature dependence of the carrier mobility provides sufficient information regarding the nature of charge carrier transport, as well as stability and performance of the device at elevated temperatures. The transfer scans measured at different temperatures for device without barrier lowering are shown in Fig. 9a. The field-effect mobility in the device is evaluated using Eq. 2, where channel length L = 9.7 µm, channel width W = ~1.5 μm, g_m_ = ∂I_D_/∂V_G_, and device capacitance C_NS_ = ε_0_ε_r_/d = 2 * 10^−4^ F/m^2^ (ε_r_ = 3.9, d = ~170 nm). Using Eq. 2, a μ_FE_ of ~5.7 cm^2^/Vs is obtained at room temperature. Figure 9b shows the variation of field-effect mobility as a function of temperature for *V*_*DS*_ of 1 V. From this data, it can be seen that higher mobility levels are consistently observed (Fig. 9b) with increasing substrate temperature until it saturates at 370 K. At 373 K, μ_FE_ increases to 19 cm^2^/Vs from its initial value of 5.7 cm^2^/Vs (room temperature). This is expected because charge carriers acquire sufficient kinetic energy at high temperatures and this results in temperature barrier lowering. However, it is interesting to note that the device show no further increase in drain current after 370 K, most likely due to the fact that the conductivity of the barrier becomes comparable to that of the semiconductor channel at high temperatures, and the device reverts to operating as a conventional TFT.

Another important SGT parameter extracted using the temperature dependent transfer scan (Fig. 9a) and output scans (data not shown) is the intrinsic gain (Av) of the present device under investigation. The Av of the SGT device has been measured at different temperatures and plotted in Fig. 9c. For the present temperature dependent stability investigations, the SGT device Av is extracted at *V*_*DS*_ = 5 V and *V*_*GS*_ = 0 V, for all temperatures.

From the data shown in Fig. 9c, no degradation in the intrinsic gain (A_V_ = *g*_*m*_/g_d_) of the SGT device is observed with increasing temperature (note that g_d_ is the output conductance). As it can be seen from Fig. 9c, the value of A_V_ first rises from 2 to 4, as the temperature increases to 343 K, and then comes back to its original value (near 2). The A_V_ curve versus temperature should in principle increase slightly with temperature as g_m_ increases faster than g_d_. However, at high temperature, when the SGT device hits the “FET” operating mode (SB at high temperature is too conductive so the channel takes over as the main current control mechanism), the value of A_V_ is supposed to drop. The obtained value of intrinsic gain using NS-SGT devices is comparatively lower than the other reported values using silicon NWs^20^. This is due to a low g_m_ value, as can be seen from the transfer characteristic. To increase the transistor gain, there are two possible solutions. Firstly, the g_m_ value can be improved by increasing the source length^28^. Secondly, the output conductance (g_d_) can be reduced by adding a field relief structure^31^. However, the second solution is not practical to implement directly on NW and/or NS structures. Nevertheless, the obtained A_V_ using SGT devices (2.1) is approximately 10 times higher than that of our NS-FET devices with ohmic contacts (0.2).

From all these temperature measurements, it can be concluded that the performances of ZnO NS-SGT devices, operating in low-field mode, are not degraded by small fluctuation in operating temperature.

## Conclusions

In conclusion, we have investigated one-dimensional (NWs) and two-dimensional (NSs) ZnO nanostructures for the realization of high performance and stable nano-transistors on conventional rigid Si/SiO_2_ substrates. Based on the statistical electrical data (collected on 8 FET devices of each device type) and the electrical gate-bias results, we can conclude that ZnO NS-FETs showed slightly superior performance compared to ZnO NW-FETs. Thereafter, ZnO NSs were used for the fabrication of source-gated transistors (SGTs). Stability tests were performed on both devices (FETs and SGTs), fabricated using ZnO NS, with respect to gate bias stress at three different operating regimes of transistors, namely off-state, on-state and sub-threshold state. Although the SGT devices showed similar *V*_*TH*_ shift trend as that of the conventional FET, SGT devices showed only 7% decrease of the on-current compared to FETs which showed more than 52% decrease of the on-current, for 2 hour operation. Based on our experimental results, we hypothesize that the on-current in the SGT is governed by the potential barrier and the depletion region at the source, hence the ON current is independent of the threshold shift. But at low V_GS_ (subthreshold), it is the channel that dictates device behavior, and it works like a conventional FET, with the expected V_TH_ shift. At last, temperature dependence of SGT performance parameters, such as effective mobility and intrinsic gain, were investigated. Resulting electrical characterization data show that SGT devices have positive temperature dependence. Moreover, the ZnO NS-SGT performances did not degrade with temperature, rather a small increase in effective device mobility and intrinsic gain of the transistor was observed. Hence, the investigated SGT devices are expected to be useful in applications where high output impedance, good current uniformity and stability are required, such as in driver transistors in emissive pixel circuits. We envisage that the present NS-SGT devices may offer practical solutions to realize high performance low-power electronic devices based on ZnO nanosheets.

## Data Availability

The raw/processed data required to reproduce these findings cannot be shared at this time as the data also forms part of an ongoing study.
